# PET/CT: Current status in India

**DOI:** 10.4103/0971-3026.43840

**Published:** 2008-11

**Authors:** Venkatesh Rangarajan, Nilendu C Purandare, Anshu R Sharma, Sneha Shah

**Affiliations:** Bioimaging Unit, Tata Memorial Center, Parel, Mumbai, 400012, India

**Keywords:** Positron emission tomography/computerized tomography, PET/CT

## Abstract

PET/CT is a new modality with integration of PET and CT systems. In India, since December 2004 there has been a steady increase in the number of imaging systems. From stand-alone PET/CT systems with on-site cyclotrons, mostly in the government sector, the modality has matured to such an extent that, today, the majority of the PET/CT scanners and cyclotrons are in the private setup; also, scanners situated in different locations (and even different cities) share the isotope produced from one cyclotron. This shows how much this field has developed and reflects the confidence of the end users. The current status of PET/CT in India is indeed healthy and heartening and the future certainly looks promising.

## Introduction

Positron emission tomography (PET) employing the radiopharmaceutical fluorine-18-fluorodeoxyglucose (FDG), an analogue of glucose that has a high rate of uptake in a wide range of tumors, is an accurate functional imaging technique in the diagnosis, staging, restaging, and therapeutic monitoring of many common cancers.[1]

FDG-PET imaging is a lengthy process from the time of isotope injection to the acquisition of fusion images. Quantification of FDG isotope uptake and distribution within the body gives a quantitative map of its distribution in the body. For accurate quantification and reproducible results, attenuation correction is needed. Image detail is also lost due to attenuation. For these reasons, attenuation correction is essential. Attenuation correction with the introduction of an onboard computerized tomography (CT) scanner-generated attenuation map is much faster than the conventional isotope-based methods.

The fused anatomic and functional image of a PET/CT study can to a great extent overcome the diagnostic dilemmas due to the physiological false positives that are often seen with stand-alone PET scanners. This feature of CT/PET has had a significant impact in oncology. Presently, no stand-alone PET scanners are being manufactured.[2,3]

## Historical Perspective

The dawn of the PET era in India dates to 30^th^ September 2002, with the inauguration of the 16-mega-electron-volt (MeV) hospital cyclotron facility and BGO -based full-ring dedicated PET scanner at the Radiation Medicine Center (Bhabha Atomic Research Center) situated at the Tata Memorial Center in Mumbai. This BGO-based full-ring dedicated stand-alone PET scanner still remains the only one of its kind in the country.

This was soon followed by a couple of hybrid gamma cameras (sodium iodide crystal–based) with a 1-inch crystal that could also double as a PET scanner, though with certain limitations. The country's first dedicated PET/CT scanner was inaugurated on December 13^th^ 2004 at the Tata Memorial Center. The year 2005 saw the inauguration of two more PET/CT facilities with on-site cyclotrons at Apollo Hospital, Hyderabad, and AIIMS, New Delhi. Presently, there are 22 functional PET scanning units in the country; 19 of these are dedicated PET/CTs and three are hybrid gamma cameras.

## The isotope

For PET/CT examination the radioisotope that constitutes the radiopharmaceutical is a positron emitter. These radioisotopes have short half-lives. The most widely used isotope, 18 Fluorine, has a half-life of 110 min. Owing to its short half-life, large quantities of the isotope are required to be ordered from the vendor to compensate for the radioactive decay.

Currently, in India, eight PET/CT facilities are supplied isotopes by their respective on-site cyclotrons. The remaining 18 are supplied by FDG suppliers from off-site cyclotrons. Two suppliers provide intercity supply of FDG. A Mumbai-based FDG supplier provides daily doses of FDG to users in Pune and also flies it to Hyderabad and Chennai.

## FDG supply

There are two types of FDG supply protocols. According to the first one, FDG is supplied at the users‘ door step; the supply is based on the number of unit doses, calibrated in such a way that there are 10 millicuries or 370 Mega Becquerel of FDG (1 unit dose) at a given time for the first dose and each dose is subsequently calibrated so that 10 millicuries are available for injection every 30 min. According to the second supply protocol, the user collects a certain quantity of FDG from the production site and pays for the dose at the time of collection.

## Ownership of the cyclotron

Currently, six out of 14 cyclotrons belong to government institutions; of the six, only one is involved in the commercial distribution of FDG. The remaining eight cyclotrons (including those being installed) are owned by private agencies. Three of these are currently functional and are supplying FDG to users.

## Issues of the cyclotron

Cyclotrons are rated on the basis of the peak power they can develop. Table 1 shows the cyclotrons grouped according to the city of location. The energy of the cyclotron (given in parenthesis) also determines the type of the isotopes that could be produced by the cyclotron.[4] According to the shielding around the cyclotron, they can be classified as self-shielded or non-self-shielded ones. The self-shielded cyclotrons are more compact than the non-self-shielded ones. The self-shielded ones form 85% of the installed cyclotrons.

**Table 1:** Medical cyclotrons in India

Location	No.	Energy of the cyclotron
Mumbai	2	Both 16 MeV
Delhi	4	Two 11 MeV; one 16 MeV; one 18 MeV
Hyderabad	2	One 11 MeV; one 16 MeV
Bangalore	1	One 16 MeV
Ahmedabad*	2	One 16 MeV; one 20 MeV
Kolkata	1	One 30 MeV
Chandigarh	1	One 16 MeV
Chennai	1	One 16 MeV
Total	14	

* Includes cyclotrons to be installed by December 2008.

Currently, 18F and 13N are the two isotopes produced for routine clinical use. 13N, as ammonia (NH3), is produced and used for myocardial perfusion PET studies. 18F FDG and 18F fluoride are the 18F-labeled PET radiopharmaceuticals available for regular clinical use; they are supplied by both the commercial and noncommercial cyclotron operators. For experimental purposes, 18F thymidine, DOPA, and mizonidazole have also been produced. Currently they are not available for commercial use. ^68^Ga is a non-cyclotron-produced PET isotope eluted from a germanium generator. The ^68^Ga obtained thus is labeled with peptides and used for oncological indications. This compound is available only from the hospital PET radiopharmacy of one center and is not available for commercial distribution.

## Regulation for cyclotron and isotope supply

The installation and operation of a cyclotron is controlled by the Atomic Energy Regulatory Board (AERB).[5] Preinstallation site approval is mandatory. The design and the facility itself, have also to be preapproved by the board before installation can commence. Following inspections, final clearance is given for beginning operations. A designated Radiation Safety Officer has overall responsibility for the safety of the personnel and the facility. With these conditions fulfilled, the facility is then licensed for regular operation.

The supply of isotope to the end user is also subject to regulatory control. The processes followed for dispatch and transport of the isotope have to meet regulatory requirements. The supplier is responsible till the isotope is handed over to the end user. In those cases where the consumer collects the isotope at the supplier's premises, the consumer is responsible for the safe transport of the isotope to the scanner site.

## Cyclotron supply issue

The time of arrival of the isotope at most on-site cyclotron centers is 9.30–11 AM. The private commercial suppliers have been able to supply FDG at the consumer site as early as 8.30 AM. Even the centers receiving isotope from private suppliers through air carrier receive their lot of isotope by 11 AM. Most centers receive isotope either as a single consignment or in two consignments. At centers with an on-site cyclotron, doses are dispatched from the cyclotron to the PET injection rooms in lots, with each lot containing enough isotope for 3–4 patients. The supply is done through a lift or chute.

## Isotope within the end user site

All PET scanner units have dedicated injection rooms and post-injection waiting rooms for patients; these rooms are specially constructed with adequate shielding as per the regulatory requirements. All centers now have the facility to handle more than one injected patient at any given time. This is needed keeping in mind the typical scan speed of scanners, which is 15–20 min per patient. Patients are imaged after a minimum waiting period of 45 min and an average waiting period of 70 min. As per regulatory requirements, the isotope is handled, stored, and disposed off in designated areas.

## Hardware issues

Table 2 enumerates the PET scanner profile in the country.

**Table 2:** PET/CT scanner profile (n = 27).

Scanner feature: PET detector crystal	Number of scanners
Bismuth germanate (BGO)	17
Lutetium oxyorthosilicate (LSO)	8
Gadalonium oxyorthosilicate (GSO)	1
Lutetium yttrium orthosilicate (LYSO)	1
**Table 2A**	
**No. of CT slices in the CT part of PET/CT**	**Number of scanners**
1	1
2	2
6	2
8	4
16	11
40	2
64	5

Others: BGO full-ring PET scanner (stand-alone PET): 1 Sodium iodide crystal-based SPECT/CT hybrid gamma camera: 5 Total number of FDG scanning instruments: 33

Except for one scanner, which is a stand-alone PET scanner, all dedicated full-ring scanners are hybrid PET/CT scanners. The hybrid device essentially has a regular CT and a regular PET scanner in a single gantry. While the earlier versions have both these strapped next to other, the later versions have true hardware integration between the CT and PET. Effectively the later versions are more compact, with a smaller tunnel. Seventeen of the scanners are of the latter type. The number of slices in the CT is the next major variable in the configuration. In India, currently, the number of slices can vary from single slice to 64 slices; this is shown in Table 2A. The CT component of the PET/CT study is used to generate an attenuation map for attenuation correction for the PET image data. Besides the above-mentioned PET/CT scanners and the stand-alone PET scanner, dual-head 1-inch sodium iodide (NaI) gamma cameras are also used as PET/CT scanners. These are regular gamma cameras with the usual 3/8-inch crystal replaced by a thicker 1-inch crystal that can also be used for PET imaging. The dedicated full-ring PET scanner as a stand-alone PET or as PET/CT has a higher sensitivity and better resolution and performance characteristics than the hybrid gamma camera. Although they are inferior in performance compared to the dedicated scanners, they can be used for performing routine nuclear medicine investigations and, moreover, the cost is much less. Currently four such camera units are in service performing PET studies.

## Imaging protocol

Following a scout, a CT scan is performed and this is then followed by the PET acquisition. The CT protocol and the number of centers practicing it is summarized in Table 3.

**Table 3:** Imaging protocols in PET/CT-current practice

Protocol	No. of centers
Non-contrast-enhanced CT (non-CECT) with nondiagnostic low-mA scan (80–110 mAs)	7
Non-CECT with low-mA scan (2.5 mAs)	4
Non-CECT with nondiagnostic low-mA scan (110 mAs) followed by regional diagnos tic CECT	3
CECT (venous phase) whole-body	10
CECT with early-phase liver, breath-hold CT at end of study	1
Non-CECT (110 mAs) with breath-hold CT of thorax	1
CECT (regional) for RT planning	5

PET scan is acquired in 2D or 3D mode, depending on the hardware. The 2D mode is reserved for large patients weighing more than 75 kg; 3D is the commoner protocol and accounts for 60% of all scans done.[6–8]

The non-contrast-enhanced low-dose CT (80–110 mAs) followed by PET is the most common protocol and accounts for nearly 70% of all PET/CT scans done in the country. The remaining 30% scans are performed in the whole-body CECT mode followed by PET acquisition. The typical scanning time in a PET/CT scanner for a whole-body scan from the skull base to the upper third of the femur is 20 min; a stand-alone PET scan takes an additional 20 min, which makes the study time a total of 40 min. The gamma camera–based PET systems take about 75 min for the same study. Cardiac PET and brain PET are performed in noncontrast CT modes.

## PET/CT work cycle

A patient after a minimum fasting period of 6 h, is injected with 5 megaBecquerel per kilogram body weight of FDG. After the uptake period, the patient empties the bladder and is taken up for the study. Oral contrast is given in 50% of the centers; the other centers ask the patient to drink water. Children are given triclofos sodium BP (an oral hypnosedative) 30 min post injection. Diazepam and furosemide are other medications that are also given, though infrequently. In three centers, general anesthesia or intravenous ketamine is used routinely in children.[9]

## Work load

The studies in government institutes outnumber those done in private centers. In a government institutional setup about 15–25 whole-body studies are done in a day over 8–10 h. In the private sector the number is 5–15, with eight per day being a good average.

PET/CT-guided biopsy or intervention and PET/CT–based radiotherapy (RT) planning are the other dimensions of the PET/CT work flow. The PET/CT data may be exported as DICOM data to the CT or the RT planning system.[10] Eight centers are currently using PET/CT for biopsy guidance and for RT planning.

## Cost of the PET scan

While it is entirely free in one center, the cost in government institutions varies between Rs. 2000 and Rs. 10000, the average being Rs. 6000. In the private sector, the cost for a whole body PET/CT scan is Rs. 15000–27000, depending on the type of protocol.

## License of PET/CT facility

A qualified nuclear physician who has been approved by the AERB becomes the primary licensee of the facility. A radiation safety officer who is a technologist approved by the AERB is delegated the responsibility of radioisotope inventory, dispensing, and disposal. The licensee periodically submits a report to the AERB. Similar to the cyclotron, the facility is set up after the plan and site have been approved by the regulator.

## Indications for PET/CT study in India

**In descending order of frequency, are as follows:**

1. Staging, assessing treatment response, restaging and follow-up of lymphoma
2. Restaging and follow-up in colorectal carcinoma
3. Staging, assessing treatment response, and follow-up of lung carcinoma
4. Restaging, follow-up, and assessing treatment response of breast carcinoma
5. Bone and soft tissue sarcomas – staging and assessing treatment response
6. Restaging, assessing treatment response, and follow-up of melanoma
7. Restaging and assessing treatment response of head and neck tumors, including thyroid tumors
8. Epilepsy – presurgery
9. Dementia, Alzheimer's
10. Cardiac viability

## Reporting the study

In most institutions the study is reported by a nuclear physician. Some institutes have, in addition, a full-time radiologist and a combined PET/CT report is given, incorporating both PET and CT findings. In many centers the CECT is reported by a part-time radiologist and this supplements the report made by the nuclear physician. Rarely, a separate CECT report and a separate PET report are also given. Ninety percent of the centers give a DICOM CD of the study along with the hard copy.

## Manpower requirement

For PET/CT departments, the regulations demand the presence of a full-time nuclear physician (MCI recognized) and an AERB-approved trained nuclear technologist. For optimum utilization of the scanner, a trained radiologist is also required. While most of the training centers for nuclear medicine have a PET facility or are in the process of acquiring one, most trained nuclear physicians and technologists are expected to have hands-on experience in PET technology. There is also a need for sensitizing radiologists to this modality during their training. New sandwich courses are needed to make radiologists competent in this hybrid modality. An apprenticeship training programme for nuclear technologists and radiographers is in operation at the Bioimaging Unit, Tata Memorial Hospital.

In the short span of 4 years after the first PET/CT was installed at the Tata Hospital there has been a rapid growth of the modality. With firm participation from the private sector the modality will grow further, and as cheaper editions of PET/CT scanners and cheaper ‘no frills’ cyclotrons become available, the cost of the modality is bound to come down.

The fusion of the hardware has surely made the nuclear medicine fraternity and the radiologists come closer to each other for a symbiotic relationship.

## References

[1] Endo K, Oriuchi N, Higuchi T, Iida Y, Hanaoka H, Miyakubo M (2006). PET and PET/CT using 18F-FDG in the diagnosis and management of cancer patients. Int J Clin Oncol.

[2] Townsend DW (2008). Dual-modality imaging: Combining anatomy and function. J Nucl Med.

[3] Blokland JA, Trindev P, Stokkel MP, Pauwels EK (2002). Positron emission tomography: A technical introduction for clinicians. Eur J Radiol.

[4] McQuade P, Rowland DJ, Lewis JS, Welch MJ (2005). Positron-emitting isotopes produced on biomedical cyclotrons. Curr Med Chem.

[5] http://www.aerb.gov.in/.

[6] Gollub MJ, Hong R, Sarasohn DM, Akhurst T (2007). Limitations of CT During PET/CT. J Nucl Med.

[7] Wong TZ, Paulson EK, Nelson RC, Patz EF EF, Coleman RE (2007). Practical approach to diagnostic CT combined with PET. AJR Am J Roentgenol.

[8] Elstrom RL, Leonard JP, Coleman M, Brown RK (2008). Combined PET and low-dose, noncontrast CT scanning obviates the need for additional diagnostic contrast-enhanced CT scans in patients undergoing staging or restaging for lymphoma. Ann Oncol.

[9] Jadvar H, Connolly LP, Fahey FH, Shulkin BL (2007). PET and PET/CT in pediatric oncology. Semin Nucl Med.

[10] Brunetti J, Caggiano A, Rosenbluth B, Vialotti C (2008). Technical aspects of positron emission tomography/computed tomography fusion planning. Semin Nucl Med.

